# Understanding Social Media Literacy: A Systematic Review of the Concept and Its Competences

**DOI:** 10.3390/ijerph19148807

**Published:** 2022-07-20

**Authors:** Karina Polanco-Levicán, Sonia Salvo-Garrido

**Affiliations:** 1 Programa de Doctorado en Ciencias Sociales, Universidad de La Frontera, Temuco 4780000, Chile; k.polanco01@ufromail.cl; 2 Departamento de Psicología, Universidad Católica de Temuco, Temuco 4780000, Chile; 3 Departamento de Matemática y Estadística, Universidad de La Frontera, Temuco 4780000, Chile

**Keywords:** social media literacy, digital literacy, media literacy, systematic literature review

## Abstract

Nowadays, people spend long periods on social media, ignoring the implications this carries in daily life. In this context, the concept of social media literacy, an emerging concept scarcely developed in the literature, is relevant. This study sought to analyze, descriptively, the main definitions and competences of the concept of social media literacy. The methodology included a systematic search of literature in the databases Web of Science, PubMed, and Scopus between 2010 and 2021, applying filters for English and Spanish, including only scientific articles. A total of 1093 articles were obtained. An article selection process took place, applying the inclusion and exclusion criteria, resulting in a total of 15 articles being selected. The findings indicate that the concept of social media literacy is based on media literacy to then integrate the characteristics and the implications of digital platforms. This is linked to the development of cognitive competences, where critical thinking, socio-emotional competences, and technical competences are fundamental, considering the social context. The development of socio-emotional competences stands out since social media are a frequent place of interaction between people.

## 1. Introduction

The transformation of society has been linked to technological changes that are an important part of people’s lives [1,2]. Digital technologies are inserted in aspects of social life, in families and relations with others, at work, in governance and political participation, and they generate new ways to shape a community [3,4,5]. In this sense, social media are widely used by different societies, transcending the geographical borders of territories and cultures, connecting the global to the local [6,7]. Staying on the Internet and social media for extended periods has resulted in media and digital literacy continuing to gain importance [1].

It is important to specify that social media differ from other types of Internet platforms in that they are characterized by their mass use, they allow content creation, and are not only consumed passively, making it possible for people who do not have formal knowledge about mass media to produce information [8]. This is even more relevant considering the cross-sectional use of social media by different age groups and that children’s exposure to cell phone screens begins at an early age [9]. Later, in adolescence they spend extensive periods on social media due to their socializing with their peers [10,11], whereas university students spend an average of 20 h a week on such digital platforms [12], it has been reported that 98.3% of survey respondents state they use social media [13]. The opposite would mean being outside a relevant social space [14]. In the older adult population, there is evidence that they use the technology less other age groups; however, the rates of social Internet use are increasing [15].

It should be noted that users are exposed to different phenomena on social media, such as publicity, images with a positivity bias, and aggressive and violent behaviors. In addition, the way in which social media operate must be considered as they use technology to filter content based on the users’ previous choice, favoring confirmation bias [16]. They also offer the opportunity to choose with whom one wishes to interact, enabling the formation of groups or communities with similar characteristics [17,18,19], which can foster negativity against what is different, which can be particularly relevant in phenomena such as cyberbullying, which has been linked to time spent on social media [20,21].

Thus, there are also messages on social media that can be potentially harmful when they are about health and personal appearance [22], considering people’s exposure to advertising and photos shared with positivity biases [23,24]. In this sense, exposure to photos that have been manipulated to achieve a positive appearance is associated with reducing body image and body satisfaction, with the increase in the desire of young women to get cosmetic surgery [25], depending on the time spent on the Internet [23].

On the other hand, users can be confronted with demands and difficulties such as the dissemination of false and manipulated news in the post-truth era [1,26], which are produced and put into circulation intentionally to obtain benefits such as more visits by users [27]. This is combined with people sharing information without a review process for this content since positive feedback from other users prevails; consequently, fake news goes viral very quickly [26]. People are needed in the role of information consumers; they must develop critical thinking, i.e., a skeptical view of the selection of the news provided through algorithms and the news sources must be tracked [4,26], since discerning veracity or falsity is a responsibility that transcends the individual [5].

It is important to note that the use of social media is not negative in itself as it can increase social capital, foster friendships and reduce feelings of loneliness; however, it depends on the user’s characteristics and how the different platforms are used [28,29]. As a result, teaching and learning competences for the use of these Internet platforms are particularly relevant since they include social and ethical aspects and technical skills [14], as well as competences that can assess information that aids in better decision-making [30].

Media literacy was defined by the Aspen Institute [31] as “the ability to sensitize, analyze and produce information for specific results” (p. 6), although this conceptualization has certainly undergone progressive transformations, moving from printed information to expression and communication that includes new symbolic forms, such as images and multimedia content. In addition, social media have enabled group collaboration and the dialogue of a large number of people who produce content [32]. It is worth noting that Hobbs [32] refers in particular to media literacy and understands it as knowledge, competences, and skills for life that make it possible to participate in today’s society by accessing, analyzing, evaluating, and creating messages in different ways and in different media, being the result of media education. For his part, Buckingham [33] emphasizes the critical component and the understanding that contents are inserted in a broad context, for example, digital capitalism. The emergence of new types of literacy is linked to the appearance of Internet and mobile communication technologies, which have resulted in the appearance of new media. Considering their impact, this is occurring with technologically based sociocultural platforms [34].

In the same vein, digital literacy refers to a broad set of competences around the use of digital media, computers, and information and communication technologies (ITC), being understood as part of other forms of literacy, such as computer, Internet, media, and informational literacy [35]. Currently, efforts are being made by the international community to guarantee digital literacy [36], because since the COVID-19 pandemic time on the Internet and social media has increased [37]. It is important to mention that digital literacy has been proposed as a strategy against social inequality, given the connection between technological exclusion and wider forms of economic and social exclusion [38], because people have fewer opportunities to develop skills due to their limited Internet connection, thereby reducing participation levels [39]. Another relevant element is that it is linked to socio-economic disadvantage with a lack of knowledge about the algorithms that these types of platforms use to recommend content [40].

Literacy in traditional and digital media is central given that we live permanently receiving messages from different sources [41]. Generally, these are focused on improving people’s competences to integrate and operate in today’s society [42]. Therefore, it is necessary to promote the development of skills such as critical thinking because even though teenagers and young adults have known the world with the Internet, they do not have better developed skills in all the areas that digital literacy addresses [43]. Nevertheless, according to Leaning [35], the difficulty arises because media literacy does not sufficiently address digital technology, considering that digital literacy does not fully develop a critical approach compared to media literacy. However, it is relevant to point out that the boundaries between the types of literacy can be blurred; in addition, other proposals progressively emerge that link different approaches such as critical digital literacy, rendering the desired distinctions complex [44,45,46].

In this sense, due to their mass use, social media have transformed the way we relate to each other, form communities, and use mass media. This has been of interest, with proposals on the issue of literacy being generated that focus particularly on these digital platforms. Therefore, Livingstone [47] indicates the need for literacy focused on social media to update the analysis of media literacy. Nevertheless, this concept has limited theoretical development and little operationalization [7,48]. In addition, there is evidence that authors define it differently; it has not been clearly established what the competences are that are included in this type of literacy given the authors working with this concept in their research.

In light of the above, this article focuses on social media literacy by performing a systematic literature review to better understand the concept in terms of the competences it provides that adequately guide efforts in the direction of teaching and learning processes in this area. The relevance of these processes must be borne in mind due to the mass use of such platforms and their use by people of different ages for extended periods, considering there are dangers in social media while at the same time they afford possibilities for interaction, entertainment, and other options that can be useful with an adequate understanding of how social media work and how to make use of them. Therefore, the aim of this study was to analyze, descriptively, the main definitions and competences of the concept of social media literacy.

## 2. Materials and Methods

**Figure 1:**
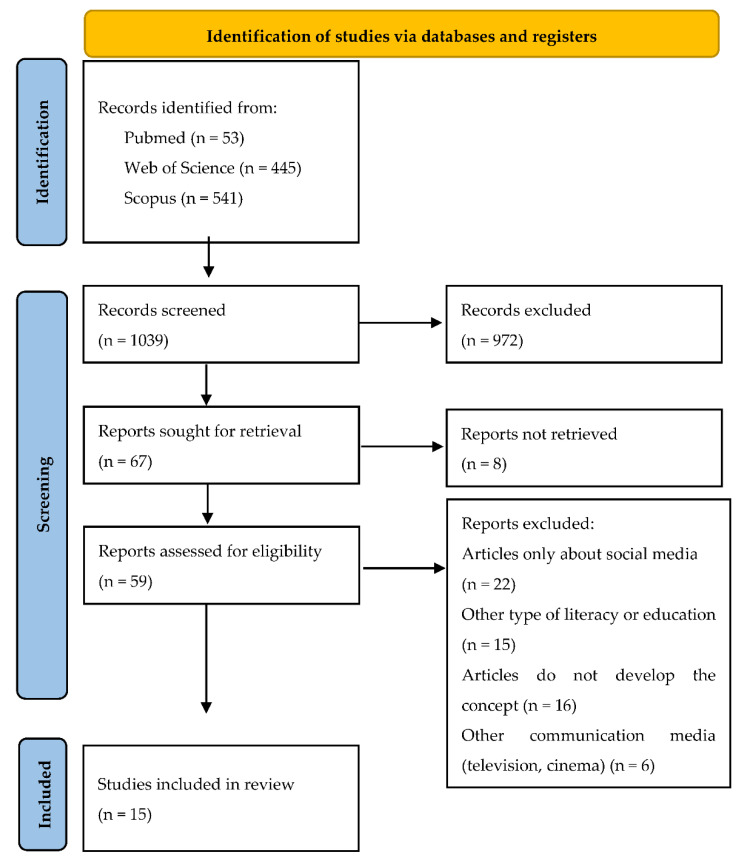
Systematic review flowchart (Adapted from Page et al., 2020 [49]).

A systematic search of the literature was done, considering the guidelines of Preferred Reporting Items for Systematic Reviews and Meta-Analyses (PRISMA) [49], in the Web of Science, PubMed, and Scopus databases in July 2021. The question that guided the search strategy was: what are the competences that must be developed to operate on social media? The search took place using free terms and terms from Medical Subject Headings (MeSH) including social media, social media sites, digital literacy, media literacy, and social media literacy. The filters were: language (English and Spanish), number of years (from 2010 to date), and article type (article). With respect to the total articles (*n* = 1039), they were first selected by relevant title, second, by relevant abstract. Then, the articles were reviewed in full (*n* = 59), and the inclusion and exclusion criteria were applied, resulting in 15 articles (Figure 1).

### 2.1. Criteria for Eligibility

Inclusion criteria: Articles were selected that proposed a conceptual definition of social media literacy and/or that demonstrated the competences that integrate this concept. Articles were included where the participants were children, teenagers, young adults, adults, and families. Only scientific articles, theoretical and empirical, in English and Spanish between 2010 and 2021 were included.

Exclusion criteria: Articles that address social media from digital literacy without specifically considering the scope of social media literacy were not included, since they do not define the concept, nor do they refer to the competences that social media literacy encompasses. In addition, articles that address digital platforms but do not consider social media were not included. Theses, conference proceedings, and systematic reviews were not included. Articles in languages other than English or Spanish or with a publication date before 2010 were also excluded.

### 2.2. Procedure

Articles were selected considering the inclusion and exclusion criteria. The articles also had to provide information that responded to the research question; therefore, those articles that did not fit as previously indicated were eliminated. Where questions or disagreements arose about the selected articles, they were resolved through the joint review by the two authors to determine their relevance and to make a decision about their inclusion.

In terms of biases of this study, the language bias was countered by including articles in Spanish and English. In terms of coverage bias, the different databases (Web of Science, Scopus, and PubMed) were reviewed.

### 2.3. Analysis Strategy

With respect to the selection final, the articles were read and reviewed completely, observing if the records provided a conceptual definition of social media literacy or if they reported on the skills that this type of literacy includes. The other criteria of inclusion and exclusion were also considered. The standard quality assessment criteria for evaluating primary research papers were also applied [50].

Later, a table was constructed to present the studies, considering first the authors, type of study, objective, and information on the sample. Then, the main results were transformed in relation to the research question to report on the studies selected and to organize the findings of this study.

In relation to the biases present in articles, generally the records describe full data in their results; moreover, the results were reported according to the analyses used, considering that this is of interest to this review.

## 3. Results

Fifteen articles were obtained for analysis from the following countries: Australia, Belgium, the Czech Republic, Germany, Indonesia, Singapore, Spain, the United Kingdom, and the United States, it being observed that interest in the concept of social media literacy is concentrated mainly in European countries that develop and contribute theoretical and empirical evidence relating to this concept (Table A1 in Appendix A).

### 3.1. Social Media Literacy: Definition

The conceptualization of social media literacy is based on media literacy [51,52,53,54,55,56,57]. However, it is emphasized that social media are oriented to the interpersonal communication that arises from the human need to establish interactions with others [48,52,53]. Thus, according to Vanwynsbergue [56], the focus would be on favoring the efficiency and efficacy of Internet communication, benefitting social relations (Table A1 in Appendix A).

On the other hand, the understanding of the particular characteristics of such platforms is worth noting, in that it is relevant how the information is presented on social media, considering the objectives after posts by both people and advertising, in addition to positivity bias [51,53,54]. Consequently, social media literacy is oriented towards the prevention of risks such as mental and physical health problems [51,53], as well as other types of consequences that can arise from interactions between people, for example cyberbullying, information spreading, and other difficulties [52,53,55].

### 3.2. Social Media Literacy: Competences

With respect to the different competences that encompass social media literacy according to the different studies, there is evidence that cognitive competences appear cross-sectionally in most of the studies searched. These include understanding, analysis, evaluation, synthesis, and the interpretation of the information, added to the assessment of the motive, purpose, realism, and credibility of the publication. Critical thinking is considered fundamental due to the large volume of information to which social media users are exposed [51,53,54,55,56,57,58,59,60,61]. In addition, according to Schreurs and Vandenbosch [54], cognitive competences include a knowledge of traditional media literacy and the dynamics of interpersonal communication on social media (Table A1 in Appendix A).

Similarly, user-generated information requires that they have knowledge of the implications of sharing personal data and the generation of information considering the digital fingerprint, since this information is used by the social media platforms and shared with other companies, so the user must evaluate what content to share [62]. Likewise, Tandoc et al. [63] report on the need to raise awareness about the content recommendation algorithms that transform the social media experience.

The technical or practical competences include the ability to create, review, organize and share contents [57,58], access, find information and use functions such as privacy settings [62], create social media accounts and publish photos and images, and make videos and memes [60,63]. These competences fulfill an important role so people of different ages can perform adequately on these digital platforms [51,55,56,57,58,59,60].

On the other hand, the socio-emotional competences are integrated by several authors into the conceptualization of social media literacy because such digital platforms are oriented to the interaction between different people who share content online; therefore, management strategies for interpersonal communications are relevant [48,51,54,55,56,63]. Festl [48] proposes that the development of social competences is central to social media literacy including participation and moral, communicative, and education competences, consistent with other studies that lend relevance to motivation, attitude, and behavior that people on social media exhibit [55,56]. In addition, Schreurs and Vandenbosch [54] note that effective competences are reflected in the use of adaptive strategies when users are exposed to difficulties on social media, as indicated in Appendix A (Table A1).

The proposals of authors that consider the relevance of the context in which social interactions occur as well as the language used on social media are worthy of note. Specifically, the differences between the different digital platforms must be taken into account since they have particular ways of operating [55]. Moreover, the sociocultural pragmatics in the different social media must be borne in mind, i.e., changes in the users’ language, relations, and behavior depending on the different social and cultural contexts that take place on the Internet [57]. This would make it possible to assess the context that could help discern veracity of the information [60], considering the increase in fake news [63].

## 4. Discussion

The objective of this study was to analyze descriptively the main definitions and competences of the concept of social media literacy. The results yielded 15 studies (Table A1 in Appendix A) that address social media literacy by either conceptualizing it, or by referring to the competences of which it consists. It should be noted that there are studies that, despite using the concept in their articles, do not develop it, or they use it to talk about another type of literacy without making a suitable distinction on the issue [22,64,65].

In relation to the findings of this study, the construction of the concept of social media literacy is based on the knowledge gained through media literacy, to then integrate elements focused on catching the particularities, characteristics, and implications of social media. In this context, it is fundamental to consider the social interactions produced on social media, the possibility of users creating content, the large amount of information that circulates on social media that includes user content and publicity from businesses, as well as the content filtering and recommendation technology. In the same vein, it is suggested that the concept of social media literacy could respond to the requirements of today’s society due to the mass and recurring use of these types of virtual platforms worldwide [47,48,51,52,53,54,55,56].

Consequently, social media literacy is an update of media literacy [47], being oriented to favoring people being able to perform adequately on social media considering the various difficulties that can arise. Without a doubt, the phenomena that occur on social media are not all negative, rather these digital platforms have benefits that could be taken advantage of better if users have greater knowledge and competences [28]. Thus, access to the benefits or opportunities that social media afford, such as the possibility of sharing with friends and relatives, should be promoted, but with strategies to protect against damaging trends or risky behaviors [54].

Generally, the analyzed studies converge in the relevance of cognitive competences in social media literacy. It is worth noting the development of critical thinking because most studies mention it being necessary to obtain a suitable understanding and assessment of the content, being aware of the reliability and credibility of the information [55,56,60], reducing the persuasive influence of mass media through the evaluation of the intention and realism of the content [53,61]. This is not an easy task due to the large volume of information and the anonymity of those who produce the content on social media [57]. In this sense, the knowledge about the algorithms with which social media work acquires relevance, presenting information to the user according to their fingerprint [40].

As Livingstone [52] points out, social media literacy is at the intersection between social and mass media, so that the relevance of socio-emotional competences stands out. The social interactions that take place between users in real time or delay time are one of the characteristics that distinguishes social media from other types of digital platforms or mass media; therefore, different authors have focused on the socio-emotional competences to conceptualize and operationalize the construct [48,51,52,53,54]. In this way, such competences can be considered a protective factor against cybervictimization [66], and a greater prosocial behavior in Internet activities is implied [67], since there are adaptive strategies against negative experiences [54].

With respect to the technical or practical competences, there is evidence that among these are the ability to access, create, review, and share content on social media, adding other functions such as those linked to privacy settings. These competences are considered in a general way; however, social media platforms are different from each other, which is why it is relevant to consider those specific skills that could help people to perform adequately on the different social media. Coincidently, Manca et al. [7] refers to a higher skill level that can be cross-sectional on the different social media and skills specific to each digital platform.

Likewise, studies have shown the relevance of the context in which the content is generated in order to assess its construction [55,57,60]. Then, the specific platform can be considered, the context in which differences in the language used and the forms of interaction between users are reflected. On the other hand, it is important to place social media within a broader social and economic context such as digital capitalism [33], being aware of the objectives of the social media companies such as generating profits [68], transforming the private experience into merchandise [69].

Another finding of this study is the different areas in which studies are being conducted that involve this concept. On the one hand, evidence shows that different authors work with this concept applied to the area of physical and mental health related to body perception [51,53,61,70], developing interventions to reduce eating disorders and the negative impact of exposure to social media because they show idealized appearances, such that social media literacy is considered a protective factor [24,61]. Meanwhile, another group of authors focuses on research with children and adolescents due to the continuous use of social media as a result of their need to establish relations with their peers and how their families mediate the use of digital platforms [48,52,54,58]. Consequently, the development of competences by teenagers is fundamental for them to operate suitably on social media, considering that parents show deficiencies in technical competences and knowledge of social media because they use them less or they use digital platforms passively [54,58].

Finally, the relevance of the analysis and the assessment of news content on social media to determine its veracity stands out in the current context [5,26,60,63]. In this sense, the contribution of social media literacy is significant since it considers aspects of such platforms, because when sharing information, it prioritizes the expectation of positive feedback from other users, or that the content supports one’s personal beliefs and values.

## 5. Conclusions

This systematic review collaborated in the understanding of the construct of social media literacy in its definition and the skills that integrate it, being considered an area of emerging research and that its development is very necessary due to people staying on social media specifically for extended periods. Social media literacy is focused on the development of different abilities that range from the technical to the socio-emotional. In this sense, social media, by making possible and favoring social interactions, bring with them requirements for people to perform adequately on digital platforms, understanding that there is no separation between the digital plane and the physical plane; therefore, a mutual influence is produced that could affect people’s experience by being exposed to the dangers on social media that worsen without the skills to deal with such situations.

On the other hand, the social, economic, cultural, and political context is integrated into the analysis conducted on social media given that such platforms have product advertising, political announcements, and other situations to which social media users are exposed. At the same time, the social media differ from each other, so it is relevant to visualize the characteristics of each of them and their differences, noting they each have their own culture that is reflected in the language, behavior, and interactions generated.

In terms of the limitations of this study, it should be noted that there may be articles that were not detected in the systematic search, or that were not selected for the analysis considering the inclusion and exclusion criteria of this study because the authors used concepts linked to media and digital literacy to refer to the concept of social media literacy. Other databases could be added to verify whether there are new articles and integrate them into the results, contributing to different research questions. Similarly, other types of articles such as systematic reviews or conference proceedings could be added since they were excluded here. With respect to the future lines of investigation, studies must be generated considering the construct of social media literacy and its relation to other constructs such as cyberbullying and cyberaggression as the dangers of social media are considered, making it possible to observe which competences that make up social media literacy are those that would mainly protect against these dangers. In addition, it would be interesting to identify the relations with constructs that reflect if social media literacy facilitate the opportunities that such platforms offer. Finally, other studies could broaden the inclusion criteria by incorporating articles that address social media literacy, although the authors have used other broader concepts or approaches in their research. This way, future studies could analyze and evaluate which of the different literacies that focus on social media obtain the best results.

## Data Availability

Not applicable.

## Appendix A

**Table A1:** Concept and competences of Social Media Literacy.

Authors	Country, Sample Age/Degree	Objective/Study Type	Results: Definition	Results: Competences
1. Daneels and Vanwynsberghe (2017) [58]	Belgium 14 parents (9 fathers/5 mothers) 35–53 years 13 adolescents (9 girls/4 boys) 12–18 years	Qualitative study (1) To examine mediation strategies defined by previous studies and their relevance for the use of social media. (2) To explore the relation between social media literacy of the parents and the choice of a certain mediation strategy.	The definition of the concept proposed by Vanwynsberghe et al., (2015) is used. These authors state they are technical and cognitive competences that users must develop so social interactions and communication on the Internet are effective and efficient.	1. Technical competences: related to the knowledge and skills to create, review, organize, produce, and share content on social media. 2. Critical cognitive competences: refer to the analysis and assessment of information and context in which it takes place considering its relevance and reliability.
2. Festl (2020) [48]	Germany 1508 students 11–18 years 66% women	Quantitative study To propose the construct of social media literacy based on skills and to develop a standardized instrument.	The concept proposed by Festl (2020) is based on the relevance that social media have to satisfy human needs such as feeling and being connected to others, especially for teenagers. This definition is based on the proposal by Pfaff-Rüdinger and Riesmeyeer (2016).	- Social competences consist of: 1. Participatory/moral competences: those related to participation without damaging others and being honest. 2. Communicative competences: refer, for example, to teenagers talking with their friends about experiences on the Internet. 3. Educational competences: related to showing others how Internet applications are used. - Each of the competences are assessed with a process-oriented perspective, i.e., considering knowledge, skills, motivation, and behavior (performance).
3. Gordon et al., (2020) [51]	Australia 700 students 11–15 years 50% men	Quantitative study To evaluate the effectiveness of a school social media literacy intervention for early adolescents.	This concept is based on media literacy, favoring understanding over how the information on social media is presented, e.g., publications by people vs. commercial enterprises. In addition, it addresses the motivations on which the selection and the way in which contents are shown are based. This is to protect against the negative impact of social media use on body image. The possibility of creating content is considered.	1. Critical thinking against the publicity on social media. Favoring the evaluation of the realism on social media to reduce the persuasion of these digital platforms. 2. Socio-emotional skills for interaction on social media. 3. Skills that make it possible to create content on social media that is positive and realistic.
4. Livingstone (2014) [52]	United Kingdom, Spain, Czech Republic. 48 participants 9–16 years	Qualitative study To introduce the concept of social media literacy. To explore the opportunities and risks that children experience on theInternet.	This concept addresses the tasks of decoding, evaluating, creating, communicating in different ways (text, image, platform, device, etc.), as well as social interaction (relations, privacy, anonymity, etc.), since these skills are integrated into the use of social media. This concept is based on media literacy and responds to the present needs of children and to the possibilities of connecting to social media, considering the positive (online opportunities) and negative consequences (risk of damage online).	
5. Livingstone (2015) [47]	United Kingdom	Theoretical study To understand the transformation of mass media and their differences with social media.	Social media literacy is understood as the update of media literacy to perform more suitable analyses of such digital platforms, since they are at the interface between “social” and “media”, which will enrich, expand, and update the important tradition of mass media education.	
6. McLean et al., (2017) [53]	Australia 101 teenage girls 13.13 years	Quantitative study To examine the effectiveness of an intervention in social media literacy on risk factors related to eating disorders in adolescents.	It is understood as integration of the media literacy and peer group theory resulting in an effective proposal for prevention.	The relevance of critical thinking in response to social media content is highlighted.
7. Newman (2015) [59]	United States	Theoretical study To address the effects of the use of Instagram on the development of identity in young adults. To propose three skills needed for social media literacy.		1. To understand the functions of Instagram: knowledge and understanding of the application and its emphasis on the artistic and visual expression of the content. 2. To evaluate and understand the authenticity of communication based on images considering the social comparison that takes place based on publications or content affecting the construction of social identity. 3. Genuine belonging: understanding that the positive feedback of other users is not necessarily related to belonging to a group.
8. Pangrazio and Cardozo-Gaibisso (2020) [62]	Australia Uruguay 276 preadolescents from 7 to 12 years	Quantitative study To identify digital practices, challenges, and consequences in preadolescents.		1. To represent digital identities in every context: to understand how the functioning of social media has implications for identity development. In addition, how digital platforms through the digital fingerprint and shared information are used to make inferences on a person’s identity. 2. To understand the implications of generating personal data: to understand that digital platforms have the power to use and distribute their users’ data with other digital companies or platforms. 3. To manage and protect the privacy in media contexts: involves understanding what content to share and with whom. Privacy management depends on the digital platform.
9. Schreurs and Vandenbosch (2020) [54]	Belgium	Theoretical study	Inasmuch as people who use social media have cognitive and affective structures that can guarantee the reduction of the risks in interactions with social media content, while they increase the benefits at the same time.	1. Cognitive structures: envisage (a) traditional media literacy; (b) characteristics of mass media; (c) dynamics of interpersonal communication on social media. 2. Affective structures: oriented to the ability to apply adaptive strategies in that than they are maladaptive when negative experiences are suffered
10. Syam and Nurrahmi (2020) [60]	Indonesia 500 students 17–24 years 46% men	Mixed method study To propose a framework of media literacy to study the critical ability of university students to process fake news on social media.		1. Competences to access social media content: to find information and use the functions. It is also relevant to understand the meaning of this content that encompasses understanding publications and the use of emoticons. 2. Competences to interpret the textual meaning of social media content: involves the ability to synthesize and critically assess the information from different social media. In the case of fake news, it offers the possibility of evaluating the credibility of the information on social media. 3. Competences to operate software: they can create, distribute, and duplicate multimedia content, i.e., gives account of the ability to create social media accounts, publish images or photos, skills to make videos and memes. 4. Competences to interpret social media content considering its context: envisages active and critical participation with regard to the information presented on social media.
11. Tamplin et al., (2018) [61]	Australia 374 participants 50% women 18–30 years	Quantitative study (1) To examine the impact of exposure to images of idealized appearance on social media on the body image of young women and men. (2) To examine social media literacy and its protective role against the negative effect of the exposure to images of idealized appearance on social media. (3) To examine whether the evaluated risk factors at the beginning of the study would moderate the effects of exposure to social media images on body satisfaction.	Understood as the knowledge and development of skills to analyze, evaluate, produce, and participate in social media, which favors critical thinking. This definition is supported by McLean, Wertheim, Masters, and Paxton (2017). Specifically, the ability to understand the motivations and techniques of companies that produce and publish commercial images and advertising, such as publications from friends and celebrity, in which the modification of images and the publication of images with a positivity bias are present.	Development of critical thinking based on the ability to assess the intent, meaning, and realism of the images and content in general on social media.
12. Tandoc et al., (2021) [63]	Singapore 3154 participants Qualitative study 62 participants 18–66 years Quantitative study 1021 participants 34.98 years (SD = 11.26) 50% women. 1000 participants 40.83 year (SD = 15.07) 52% women 1071 participants 40.39 year (SD = 12.26) 50% men	Mixed method study To examine which competences social media users require to avoid problems on social media.		1. Technical competences: involves knowing how to create or delete an account, how to add friends and how to publish information. 2. Privacy and algorithmic awareness: need to protect personal information or content posted on social media platforms, for which it would be relevant to know the privacy settings and limit what it is published. It also involves awareness about how private data are used to modify the experience on social media. Thus, critical thinking competences are necessary. 3. Management of social relations: linked to the management strategies of interpersonal communication. They may also be associated with technical competences, for example, when the friends’ network has to be segmented so certain publications are hidden from some people. 4. Informational awareness: refers to the competences to distinguish between information and accounts that can be true or false.
13. Vanwynsberghe and Verdegem (2013) [55]	Belgium	Theoretical study To propose a multidimensional framework to integrate social media literacy in an education environment.	It is understood as the practical, cognitive, and affective competences required to access, analyze, evaluate, and create content on social media in a variety of contexts. In addition, the understanding of the implications of the participatory culture on social media is contemplated, which considers: (1) using and applying media literacy in the participatory culture generated on social media; (2) visualizing and contemplating the differences among the different social media; (3) being aware of the change from passive consumption to users who are active in content creation.	Conceptual proposal that consists of three competences and sub-competences: 1. Cognitive competences: considers the knowledge and critical thinking to analyze and evaluate social media. 2. Practical competences: includes the possibility of creating content on social media, also involves looking for, opening, and reading information on social media. 3. Affective competences: considers motivational disposition and self-efficacy. It also alludes to the possibilities of communicating adequately with other people through social media. In addition: 4. The interaction between the consequences related to these three activities, including the understanding of the dissemination of personal information and the commodification present on social media.
14. Vanwynsberghe et al. (2015) [56]	Belgium 184 librarians 73.5% women. 24 to 63 years (46.28 years; SD = 9.75)	Quantitative study To identify the profiles of librarians in relation to social media literacy.	The definition by Vanwynsberghe and Verdegem, 2013 is used, considering the development of competences and the motivation to interact and communicate effectively and appropriately.	1. Cognitive competences: alludes to the critical analysis and evaluation of motives and objectives behind the consumed contents, the language of the messages, and the context in which the content is produced. 2. Affective competences: refers to the motivation and attitude to social media manifested in the assessment of social media and the behavior displayed. 3. Practical competences: envisage access and knowledge about how social media work. The authors refer to these competences as “knowledge of the buttons”.
15. Yeh and Swinehart (2020) [57]	United States 66 students 51.5% women. 18–21 years	Mixed method study To examine the characteristics and trends of social media use by students of English.	This study uses the definition by Vanwynsberghe et al. (2015) in relation to social media literacy.	1. Technical competences: it includes how to access, create, navigate, organize, and share content on social media considering the distribution and design specific to each platform. 2. Cognitive competences: refer to understanding, evaluating, and critically analyzing social media content considering its context, application, and credibility. It also includes the information overload that leads to difficulties in evaluating it, particularly considering anonymity. 3. Sociocultural pragmatics of online environments: This refers to the change that occurs in the language, interaction, and behavior as part of different social and cultural contexts formed online. Specifically, in this study the informal use of the language is considered relevant.
